# Supplementary material for: Patient-specific genomics and cross-species functional analysis implicate LRP2 in hypoplastic left heart syndrome
Source: eLife. 2020 Oct 2;9:e59554. doi: 10.7554/eLife.59554 (PMC7581429; doi:10.7554/eLife.59554)
Supplement: Supplementary file 8. [file elife-59554-supp8.pdf]

Supplemental Table 8. Case-Control Association Analysis of Rare Variants in *LRP2*

	CAVA	HLHS (n=117)			Controls (n=861)			p_value
		Total SNVs	Total Subjects		Total SNVs	Total Subjects		
Total number of variants (no predicted-damaging threshold)	All	1568	117	100.00%	10772	861	100.00%	0.0035
Missense	NSY	31	29	24.80%	147	142	16.50%	0.0178
Splice Region Intron	SS	2	2	1.70%	19	19	2.20%	0.2206
Splice Region: Inframe, Missense, Synonymous	EE	2	2	1.70%	16	16	1.90%	1
Synonymous	SY	15	14	12.00%	83	79	9.20%	0.1545
1 KB Upstream	N/A	4	4	3.40%	18	18	2.10%	0.2976
5' untranslated region	5PU	0	0	0.00%	1	1	0.10%	0.5567
3' untranslated region	3PU	10	7	6.00%	38	37	4.30%	0.0934
Intron	INT	1504	117	100.00%	10450	861	100.00%	0.0082
Variant lies within an within active histone marks from CHIPSeq data confined to human cardiovascular tissue (n=21)	N/A	53	39	33.30%	374	285	33.10%	0.6301
Variant lies within an within active histone marks from CHIPSeq data confined to human fetal heart (n=3)	N/A	25	22	18.80%	158	149	17.30%	0.5692
Variant lies within a transcription factor binding site	N/A	94	60	51.30%	590	394	45.80%	0.1847
