# Supplementary material for: Patient-specific genomics and cross-species functional analysis implicate LRP2 in hypoplastic left heart syndrome
Source: eLife. 2020 Oct 2;9:e59554. doi: 10.7554/eLife.59554 (PMC7581429; doi:10.7554/eLife.59554)
Supplement: Transparent reporting form [file elife-59554-transrepform.pdf]

eLIFE
1st Floor
24 Hills Road
Cambridge CB2 1JP, UK
P 01223 855340
W elifesciences.org
T @elife

eLife's transparent reporting form

We encourage authors to provide detailed information *within their submission* to facilitate the interpretation and replication of experiments. Authors can upload supporting documentation to indicate the use of appropriate reporting guidelines for health-related research (see EQUATOR Network), life science research (see the BioSharing Information Resource), or the ARRIVE guidelines for reporting work involving animal research. Where applicable, authors should refer to any relevant reporting standards documents in this form.

If you have any questions, please consult our Journal Policies and/or contact us: editorial@elifesciences.org.

Sample-size estimation

- You should state whether an appropriate sample size was computed when the study was being designed
- You should state the statistical method of sample size computation and any required assumptions
- If no explicit power analysis was used, you should describe how you decided what sample (replicate) size (number) to use

Please outline where this information can be found within the submission (e.g., sections or figure legends), or explain why this information doesn't apply to your submission:

No power calculations were performed for the single-family candidate gene discovery and rare variant burden testing aspects of the study. For burden testing, the number of controls versus cases was >6:1, exceeding the typical ratio of 2-3:1 for such analyses, and *P* values were calculated after correcting for multiple testing.

In our preliminary high-throughput screening studies we calculated the Z' using 3, 4, 6, and 8 wells. 4 wells were the smallest number of wells to achieve a Z' > 0.7. Therefore, for all the subsequent experiments at least 4 wells were used.

For Drosophila hearts, we calculated a power > 0.9 for N > 15 samples.

Replicates

- You should report how often each experiment was performed
- You should include a definition of biological versus technical replication
- The data obtained should be provided and sufficient information should be provided to indicate the number of independent biological and/or technical replicates
- If you encountered any outliers, you should describe how these were handled
- Criteria for exclusion/inclusion of data should be clearly stated
- High-throughput sequence data should be uploaded before submission, with a private link for reviewers provided (these are available from both GEO and ArrayExpress)

Please outline where this information can be found within the submission (e.g., sections or figure legends), or explain why this information doesn't apply to your submission:

All candidate variants and genes identified from whole genome and RNA sequencing data are annotated in supplemental tables.
For iPSC data, each experiment was repeated at least 3 times in independent biological replicates. All information is included in "Material and Method" sections, in the Figure legends and in the Results sections. A Student's t-test was used when comparing two conditions. One-way ANOVA was used when comparing more than two conditions. Values were considered significantly different when $p<0.05$.

For fly data, if possible 2 RNAi lines were used for each experiment (1 GD, 1 KK line).

For all experiments no data was excluded.

Statistical reporting

- Statistical analysis methods should be described and justified
- Raw data should be presented in figures whenever informative to do so (typically when N per group is less than 10)
- For each experiment, you should identify the statistical tests used, exact values of N, definitions of center, methods of multiple test correction, and dispersion and precision measures (e.g., mean, median, SD, SEM, confidence intervals; and, for the major substantive results, a measure of effect size (e.g., Pearson's r, Cohen's d)
- Report exact p-values wherever possible alongside the summary statistics and 95% confidence intervals. These should be reported for all key questions and not only when the p-value is less than 0.05.

Please outline where this information can be found within the submission (e.g., sections or figure legends), or explain why this information doesn't apply to your submission:

For rare variant burden testing, details of statistical methodology are provided in the supplemental methods.
For iPSC data, Student's t-test and One-way ANOVA were used. Data in the figures were represented using bars and errors bars. Bars indicate mean and errors bars indicate standard deviation.
For fly data, Wilcoxon rank sum test was used. Data is shown as box and whisker plots, with median, outliers and +/- 1.5x IQR.

(For large datasets, or papers with a very large number of statistical tests, you may upload a single table file with tests, Ns, etc., with reference to sections in the manuscript.)

Group allocation

- Indicate how samples were allocated into experimental groups (in the case of clinical studies, please specify allocation to treatment method); if randomization was used, please also state if restricted randomization was applied
- Indicate if masking was used during group allocation, data collection and/or data analysis

Please outline where this information can be found within the submission (e.g., sections or figure legends), or explain why this information doesn't apply to your submission:

Diagnosis of HLHS was based on established echocardiographic criteria. Mayo Clinic Biobank control subjects were selected after excluding individuals with diagnostic codes for personal or family history of congenital heart disease.

All fly experiments were done double-blinded.

Additional data files ("source data")

- We encourage you to upload relevant additional data files, such as numerical data that are represented as a graph in a figure, or as a summary table
- Where provided, these should be in the most useful format, and they can be uploaded as "Source data" files linked to a main figure or table
- Include model definition files including the full list of parameters used
- Include code used for data analysis (e.g., R, MatLab)
- Avoid stating that data files are "available upon request"

Please indicate the figures or tables for which source data files have been provided:

eLIFE
1st Floor
24 Hills Road
Cambridge CB2 1JP, UK
P 01223 855340
W elifesciences.org
T @elife

Human genetics source data are provided in supplemental tables.

eLife Sciences Publications, Ltd is a limited liability non-profit non-stock corporation incorporated in the State of Delaware, USA, with company number 5030732, and is registered in the UK with company number FC030576 and branch number BR015634 at the address 1st Floor, 24 Hills Road, Cambridge CB2 1JP | August 2014
